# Impacts of pragmatic implementation science in a primary care laboratory

**DOI:** 10.1017/cts.2024.682

**Published:** 2024-12-18

**Authors:** Nathalie Huguet, Sonja Likumahuwa-Ackman, Heather Holderness, April Lee, Jennifer E. DeVoe

**Affiliations:** 1 Department of Family Medicine, Oregon Health & Science University, Portland, OR, USA; 2 OCHIN Inc., Portland, OR, USA

**Keywords:** Implementation science, community-engaged research, primary care, translational science, research training

## Abstract

The Implementation Science Centers in Cancer Control (ISC3) initiative, funded by the National Cancer Institute, called for the development of implementation laboratories to bolster implementation science, create research-ready environments, and expedite adoption and implementation of evidence-based interventions (EBIs) into practice. The Building Research in Implementation and Dissemination to close Gaps and achieve Equity in Cancer Control (BRIDGE-C2) Center is one of seven ISC3 centers. BRIDGE-C2 aims to identify strategies to improve implementation of cancer prevention EBIs and conduct research / develop pragmatic methods to tailor, enhance, and support the adoption and sustainability of these strategies; advance implementation science; and build capacity and training opportunities. Since its inception, the BRIDGE-C2 Center has been conducting research and training activities to advance knowledge on how to effectively implement strategies to improve cancer prevention EBIs in primary care clinics serving socioeconomically disadvantaged patients. The translational science benefits model (TSBM) provides a useful framework for organizing a description of the BRIDGE-C2 Center’s activities. In this paper, we describe examples of BRIDGE-C2 activities and the specific impact indicators within each relevant domain/subdomain of the TSBM, demonstrating that a single activity or project has multiple impacts on methods and capacity building, clinical domains, and community health.

## Introduction

Effective implementation starts with partnerships to understand priorities and adequately target needs, enrich perspectives and learnings, to understand and adapt to context, and to build capacity and engagement in implementation activities. Calls have been answered to develop implementation laboratories to avoid research waste [1], create research-ready environments, and expedite adoption of evidence-based practices. An implementation laboratory is modeled after learning health systems or practice-based research networks [1]. It is a research-ready environment comprised of practitioners and other stakeholders, partnered with researchers, in which to test implementation strategies. The National Cancer Institute (NCI) funded seven Implementation Science Centers in Cancer Control (ISC3) with the goal of building implementation laboratories, advancing implementation science, and improving adoption of evidence-based interventions (EBIs) in cancer control [2–5].

The Building Research in Implementation and Dissemination to close Gaps and achieve Equity in Cancer Control Center (BRIDGE-C2) was one of the seven centers funded by NCI’s ISC3 initiative. BRIDGE-C2 builds on a longstanding partnership between the Department of Family Medicine at Oregon Health & Science University (OHSU) and the OCHIN practice-based research network (PBRN) of community health centers (CHCs). BRIDGE-C2 is a community-academic partnership designed to discover innovative strategies to improve the implementation of cancer prevention EBIs for socioeconomically disadvantaged populations and support advancements in the field of implementation science.

BRIDGE-C2 advances knowledge regarding how to effectively implement strategies to improve EBIs in CHC settings that can be spread to many other primary care settings delivering care to large populations of people. CHCs provide excellent care to over 30 million people [6]. By reducing barriers to the cost for medical care, CHCs deliver services to populations who have been marginalized and disadvantaged, such as patients experiencing homelessness, undocumented immigrants, and non-English speakers. A substantial proportion of CHCs patients have low income, with over 90% reporting incomes ≤150% of the federal poverty level. Further, a large proportion of CHC patients do not have health insurance or are Medicaid beneficiaries and are patients with multimorbidity [6]. Like many primary care practices serving populations impacted by health inequities, CHCs face multilevel barriers to ensuring that their patients receive EBIs in a manner equitable to patients in better-resourced care settings. CHCs offer a generalizable window into the feasibility, success, and sustainability of strategies that seek to implement cancer prevention EBIs [7]. To overcome implementation challenges, barriers must be better understood, and effective implementation strategies and methodologies must be identified. BRIDGE-C2 was established to (1) identify strategies to improve implementation of cancer prevention EBIs in primary care settings and conduct research/develop pragmatic methods to tailor, enhance, and support the adoption and sustainability of these strategies; (2) advance implementation science; and (3) build capacity and training opportunities.

We sought to understand and measure the impacts of BRIDGE-C2 activities across those three aims. To do this, we utilized an adapted translational science benefits model (TSBM) [8]. Developed at the Institute of Clinical and Translational Sciences (ICTS) at the School of Medicine and the Brown School at Washington University in St. Louis, Missouri, United States, the TSBM is a framework of 30 indicators across four domains (clinical, community, economic, and policy) that are designed to measure the actual and/or potential impact of public health and clinical research in a variety of settings. The adapted TSBM was created through a process through the ISC3 network to adapt the TSBM to add implementation science impact indicators (Supplemental Table 1). The process and adapted TSBM are described fully in Emmons et al and Cuevas Soulette *et al*. [9,10]. This study maps the BRIDGE-C2 Center activities to the adapted TSBM to demonstrate the utility of the TSBM in understanding and measuring the impact of the Center’s activities.

## BRIDGE-C2 implementation laboratory

BRIDGE-C2’s Implementation Laboratory spans two networks and encompasses 808 clinics in 22 states. The Implementation Laboratory is highly diverse and represents “real world” healthcare delivery settings with teams caring for populations experiencing some of the greatest health inequities. Within each network, the clinics share a linked Epic© electronic health record (EHR); data from each network’s EHR enable surveillance of cancer screening and prevention care services and other relevant indicators across and between the networks. In addition, clinicians and patients within this Implementation Laboratory participate in and assist with directing the research conducted through existing stakeholder groups.

OCHIN, Inc. became a 501(c)(3) nonprofit organization in 2004, when a collaborative of CHCs came together with a common set of health information technology (HIT) needs, and a shared goal of ensuring quality healthcare services supported by state-of-the-art HIT capabilities for underserved and vulnerable populations. OCHIN offers a fully hosted, highly customized instance of OCHIN Epic practice management and EHR solutions to its member CHCs. OCHIN also offers central support to help members implement practice changes, including coaches, trainers, and an online training library. The term CHC encompasses many types of centers, including federally qualified health centers (FQHCs), FQHC look-alikes, rural health centers, school-based health centers, and behavioral and dental clinics co-located with primary care. CHCs vary in size, location, staff roles, clinical workflows, and patient populations. This network offers diversity in clinics and patient populations in multiple states across the network. The large number of clinics allows for large-scale implementation science trials in primary care settings.

OHSU Health is a network of 12 academic primary care clinics in the Portland, Oregon metropolitan area. The network is comprised of clinics providing family medicine, general internal medicine, and general pediatrics services; and as an academic medical center, OHSU Health also has medical residents and other learners. OHSU Health primary care clinics represent a spectrum of healthcare delivery settings (e.g., an FQHC, a rural health center, and a school-based health center). All OHSU Health primary care clinics share a linked Epic© EHR and are connected to OHSU’s regional network of hospital services. This integrated network, along with the different levels of learners, permits smaller-scale experimental studies to be developed and tested for potential large-scale deployment. In addition, OHSU patients, students, residents, and clinicians participate in and help direct the research and scholarship work conducted in these clinics. The OHSU Department of Family Medicine provides implementation support to practices, health information technology (HIT) developers who can build tools in the EHR to promote implementation, trainers and educators, and audit and feedback data. The network’s clinics have 467 providers located across different communities, 85,000 unique patients with at least one visit in 2022, and more than 240,000 patient visits in 2022 across the sites [11].

BRIDGE-C2 is organized to solidify a tight connection between OHSU and OCHIN that will enable each of these unique organizations to teach and learn from each other in powerful ways that would not be possible without this unifying infrastructure. BRIDGE-C2 creates bidirectional approaches to educating and learning from CHC teams, community partners, and scientists. BRIDGE-C2 engages members of the laboratories to set research priority, identify care gaps, select research pilots, participate in proposing and conducting research pilots, and in dissemination activities. Engaging clinical partners from initial concept phase of proposed pilots to dissemination ensures feasibility and promotes sustainability of strategies developed to improve delivery of cancer preventive care.

## Impacts using the adapted translational science benefits model

Engaging our community and academic partners, the BRIDGE-C2’s team reviewed and matched each activity and pilot conducted in the Center to the TSBM domains. The team identified three domains and seven subdomains best fitting the Center’s activities: Domain 1: Implementation Science Field; Domain 2: Clinical; and Domain 3: Community. We describe examples of activities within each of our three focus areas (research, engagement, and training) and the specific impact indicators within each TSBM domain/subdomain identified. In many cases, activities spanned across multiple TSBM domains (Tables 1, 2 and 3). The team reviewed each completed or in-progress pilot, engagement, and capacity-building activity and determined whether these activities fit the TSBM impact indicators. To identify the indicators, the team assessed (1) the innovation of the activity relative to the implementation science field (e.g., novel method), (2) the results of the studies and associated clinical impact, and (3) the variability in engagement and learning activities and level and types of trainees. Further details about all BRIDGE-C2 Center activities can be found on the Center’s website (www.bridgetoinnovation.org).

**Table 1.** BRIDGE-C2 activities organized by the implementation science domain of the TSBM

	BRIDGE-C2 Center Activities															
**TSBM Indicators** • actual impact ◇ potential impact	Pilot: Methods to predict adoption/sustainability of EHR tools	Pilot: EHR proficiency and efficiency among CHC clinicians	Pilot: Methods for tracking adaptation to implementation support strategies	Toolkit: COVID Vaccination	Toolkit: Pragmatic guide for implementation of social risk referral-making and documentation	Pilot: Evaluation of use of E-referral to Quitline for smoking cessation	Pilot: Social risk and up-to-date status of colorectal breast cervical cancer screening and test	Pilot: Impact of COVID on cancer-related care delivery in CHCs	Pilot: Cancer prevention among transgender and gender diverse patients	Pilot: Evaluation of communication process of EHR changes	Pilot: Intervention to improve melanoma screening in primary care	Pilot: Evaluating adoption and adapting a Cervical Cancer Cytology Tool	Pilot: Evaluate adoption of a batch preventive care order EHR tool to determine adaption needs	Course: Introduction to Implementation Science	Mentoring	Practice surveillance
Subdomain: Implementation Science Methods and Measures																
Developed new measures of implementation determinants, processes, or outcomes		•	•													
Developed new methods for implementation strategy selection and optimization, or for identifying and prioritizing implementation determinants	•	•	•							•						
Identified gaps in the literature	•	•	•		•				•			•				
Rapid needs assessment								•			•	•	•			•
Used rapid cycle testing designs				•	•			•								
Developed or adapted an implementation process or strategy with an explicit focus on health equity				•	•						•	•	•			
Subdomain: Capacity-Building																
Partner led or participated in grants, publications, and presentations	•	•	•	•	•	•	•	•	•	•	•	•	•		•	•
Developed strategy for return of results to research partners and beyond; strategy is preferred by partner, relevant and actionable		•		•	•					•		•	•		•	•
Increased skills of mentors	•				•				•		•	•	•	•	•	
Increased skills of early investigators and trainees at all levels	•	•	•		•			•	•		•	•	•	•	•	•
Increased diversity of investigator teams		•						•	•			•	•		•	
Included early investigators and trainees	•	•	•	•	•		•	•	•		•	•	•	•	•	
Developed/Refined tools to aid in the planning of IS projects, selection, combination, adaptation, use and assessment of IS TMFs	•				•											•
Developed partner skills in implementation processes			•	•	•			•			•	◇	◇	◇	•	•

*Note:* EHR, electronic health record; TSBM, translational science benefits model; IS, Implementation Science; TMF, theories, models, and frameworks.

**Table 2.** BRIDGE-C2 activities organized by the clinical domain of the TSBM

	BRIDGE-C2 Center Activities															
**TSBM Indicators** • actual impact ◇ potential impact	Pilot: Methods to predict adoption/sustainability of EHR tools	Pilot: EHR proficiency and efficiency among CHC clinicians	Pilot: Methods for tracking adaptation to implementation support strategies	Toolkit: COVID Vaccination in Primary Care	Toolkit: Pragmatic guide for implementation of social risk referral-making and documentation	Pilot: Evaluation of use of E-referral to Quitline for smoking cessation	Pilot: Social risk and up-to-date status of colorectal breast cervical cancer screening and test	Pilot: Impact of COVID on cancer-related care delivery in CHCs	Pilot: Cancer prevention among transgender and gender diverse patients	Pilot: Evaluation of communication process of EHR changes	Pilot: Intervention to improve melanoma screening in primary care	Pilot: Evaluating adoption and adapting a Cervical Cancer Cytology Tool	Pilot: Evaluate adoption of a batch preventive care order EHR tool to determine adaption needs	Course: Introduction to Implementation Science	Mentoring	Practice surveillance
Subdomain: Procedures/Guidelines																
Help i-Lab partners to address guideline changes or new discoveries in terms of additional clinical services needed by patients				•				◇		◇	•	•	•		•	◇
Documented the impact of efforts to help partners address guideline recommendations						•	•		•			•	•			•
Develop treatment and implementation manual				•	•											•
Reduced use of low value clinical practices overall and among patient who experience inequities												•	•			
Subdomain: Tools and Products																
Used technology to increase implementation of EBIs and/or to reduce inequities	◇					•				◇	•	•	•			
Developed/refined and implemented software infrastructure to deliver test results related to EBI												•	•			
Used technology to track the uptake and sustainment of EBIs and/or implementation strategies					◇	•						•	•			•
Workflow development related to EBI, or other support to help with implementation of high value cancer prevention and control activities/services					•	•	•				•			◇	◇	◇

*Note:* EHR, electronic health record; TSBM, translational science benefits model; i-Lab, Implementation Laboratory; EBI, evidence-based Intervention.

**Table 3.** BRIDGE-C2 activities organized by the community domain of the TSBM

	BRIDGE-C2 Center Activities															
**TSBM Indicators** • actual impact ◇ potential impact	Pilot: Methods to predict adoption/sustainability of EHR tools	Pilot: EHR proficiency and efficiency among CHC clinicians	Pilot: Methods for tracking adaptation to implementation support strategies	Toolkit: COVID Vaccination in Primary Care	Toolkit: Pragmatic guide for implementation of social risk referral-making and documentation	Pilot: Evaluation of use of E-referral to Quitline for smoking cessation	Pilot: Social risk and up-to-date status of colorectal breast cervical cancer screening and test	Pilot: Impact of COVID on cancer-related care delivery in CHCs	Pilot: Cancer prevention among transgender and gender diverse patients	Pilot: Evaluation of communication process of EHR changes	Pilot: Intervention to improve melanoma screening in primary care	Pilot: Evaluating adoption and adapting a Cervical Cancer Cytology Tool	Pilot: Evaluate adoption of a batch preventive care order EHR tool to determine adaption needs	Course: Introduction to Implementation Science	Mentoring	Practice surveillance
Subdomain: Health care delivery, health activities, and products																
Increased testing/screening in target setting	◇	◇				•	◇		◇	◇	◇	•	•			
Developed and/or test different training strategies											•					
Provided training to clinical service providers						•					•	•		•	•	
Increased access to care (e.g., telehealth) overall, and among groups experiencing inequities; documented impact on access and inequities	◇	◇				◇	◇		◇	◇	◇	◇	◇			
Sustained use of EBIs and/or implementation strategies in partner organizations that participated in pilots	◇				◇						◇	•	•			
Developed new implementation support resources for clinics				•	•					•						
Created a How-To guide for implementation in primary care clinics and community settings				•	•						•	•				•
Subdomain: Health Care Characteristics																
Tested interventions to improve access, delivery, quality of cancer screening, and prevention (e.g., tested transportation options to cancer screening center)						•					•	•	•			
Developed new delivery channels (e.g., mobile services, telehealth); documented improvement in equity or at least no worsening											•					
Identified implementation strategies that are most effective at increasing the uptake of the EBI and at reducing inequities	◇				◇		◇	•	◇		•	•	•	•		◇
Subdomain: Health Promotion																
Increased uptake of EBI leads to Improved markers of health and/or reduced inequities	◇					◇			◇		◇	◇	◇			
Increased access to treatment for preventable diseases, overall and/or by groups experiencing inequities	◇				•	◇	◇		◇		◇	◇	◇			◇

*Note:* EHR, electronic health record; TSBM, translational science benefits model; EBI, evidence-based Intervention.

## Domain 1: Implementation Science

An objective of the ISC3 initiative was to advance implementation with an emphasis on addressing equity. BRIDGE-C2’s approach to achieving this objective has been focusing methods on advancement specific to adoption, sustainability, and adaptation tracking and building capacity in implementation science. Our work has impacts across two subdomains: Implementation science methods and measures and capacity building (Table 1).

### Subdomain 1a: Implementation science methods and measures

The Methods and Measures subdomain includes five categories of indicators: measures development, methods development, use of rapid cycle/data collection strategies, adaptation, and developed methods for examining clinical/community partner data in new ways/formats that supports their work. BRIDGE-C2 conducted a wide range of pilots [12–14] that advance methods and our understanding of adaptation, adoption, and sustainability. Through this work, we had impacts in all five categories of indicators.

For example, the BRIDGE-C2 “Precision Implementation” pilot developed and validated a novel prediction model of adoption and sustained use of an EHR-related tool, a common implementation strategy. This pilot used machine learning to assess the performance of 25 EHR clinic-level indicators in predicting uptake of the tools. Although prediction algorithms are imperfect and cannot replace working directly with clinics, this novel approach is useful for resource planning and sampling decisions (i.e., which clinics are unlikely to implement an intervention). This pilot is an important step toward efficiently tailoring and deploying implementation support strategies for information technology innovations.

For another example, a significant strength of the Center and partnership with the implementation laboratory is the ability to do rapid assessment, testing, and evaluation. Acceleration of the adoption and equitable deployment of evidence-based cancer prevention, early detection, and control strategies was highlighted by the NCI Cancer Moonshot^SM^ Blue Ribbon Panel [15]. The ISC3 initiative [3] was created to advance this vision of rapid development, testing and refinement of innovative interventions, using a unique pilot-study based research model that emphasizes collaboration and speed translation into practice. A good example of our rapid response is our COVID-related research which had impact on the TSBM indicators “rapid needs assessment” and “used rapid cycle testing designs.” Due to BRIDGE-C2’s robust surveillance data infrastructure, we were able to rapidly extract EHR data and interview clinicians and clinical leaders to understand how the BRIDGE-C2’s Implementation Laboratory adapted and changed care delivery. In March 2020, CHCs increased monthly telemedicine visits from 4.6/1000 patients to 436/1000 patients [16]. We also saw large decreases (85% decline) in cancer preventive procedures and orders at the onset of the pandemic and a slow return to baseline [17]. We learned about the importance of leadership support (such as providing training and technical support for telemedicine), quality improvement capacity, and processes for patient outreach were critical elements for CHCs to quickly adapt to the pandemic [17]. These rapid assessment learnings (e.g., how practices adapted to ensure delivery of cancer preventive care, what tools they implemented and developed, how they supported adoption of these tools, what they de-implemented, and how they overcame barriers) are having an impact on the field of implementation science.

### Subdomain 1b: Capacity building

This subdomain includes three categories of indicators: building partner/practitioner research capacity, engagement, and build implementation science research capacity. BRIDGE-C2 greatly emphasized multifaceted capacity building. BRIDGE-C2 developed and coordinated career development resources, and opportunities for mentoring, and connections to existing institutional and national programs for early career and mid-career investigators. BRIDGE-C2 focused on three specific capacity building activities: implementation science research capacity, research capacity among partners, and career development for investigators, having significant actual impacts across all three categories within this subdomain.

First, BRIDGE-C2 increased implementation science research capacity by developing and offering a highly successful six-week “Introduction to Implementation Science” course as part of the OHSU Human Investigations Program (HIP), a program of the Oregon Clinical and Translational Research Institute, which offers an integrated clinical and translational research education curriculum. BRIDGE-C2 investigators and staff prepared an interactive curriculum for medical students, residents, fellows, and faculty learners who are expanding their knowledge of clinical research. To date, 65 participants have finished the course and prepared posters describing an implementation science project. This course has measurable impacts on several indicators within the capacity building subdomain.

Second, BRIDGE-C2 supported mentoring, which led to increasing skills of early investigators and trainees at all levels, increasing diversity of investigator teams, and including early investigators and trainees in research. BRIDGE-C2 mentees included 19 community partners and 71 learners at different levels (e.g., high school students, medical students, post-doctoral students, and faculty). The mentees had different career trajectories and impact. For example, three clinician faculty members were mentored in pilot research. On one pilot, focusing on implementing a toolkit to improve melanoma screening in primary care, two clinical faculty members co-led the team, paired with a research faculty. One of these co-leads, a family physician, noted, *“As busy clinicians with aspirations of conducting more research, the support of the BRIDGE-C2 has been invaluable. The BRIDGE-C2 team has offered expertise in practiced-based research that was lacking in previous iterations of our study and having the project management team helps us understand the hurdles we need to clear to get the study off the ground. They help us set realist goals and have been tremendous at keeping us on track. We have received invaluable assistance with the IRB application and have learned it’s extremely important to have a person with experience in that role on the team. Being part of a multidisciplinary team with a wide range of skills has been invaluable for conceptualizing and implementing our melanoma pilot” [18]*.

As another example, BRIDGE-C2 received a diversity supplement to provide mentored research training to a post-master’s degree fellow from a disadvantaged background. The fellow received scientific mentorship on research, leading one pilot and its dissemination. He received professional mentorship including CV development and communication and was provided frequent informal and structured feedback. He attended two conferences and met with eight clinical faculty for career discussions. He shadowed three clinicians in two departments. The fellow matriculated to medical school in August 2023. He stated, *“As an aspiring physician and public health researcher, I have found the mentorship and support BRIDGE-C2 has given me to be invaluable. Since I come from a socioeconomically disadvantaged background, there have been many times when I did not know if I was capable of achieving my career goals. By working with BRIDGE-C2 faculty and meeting different mentors as an NIH diversity fellow, I now have more confidence that I will be able to succeed and give back to the communities I deeply care for” [19].*

Third, BRIDGE-C2 increased partners’ skills in implementation science and helped develop and refine tools. A principle of BRIDGE-C2 is to foster bidirectional engagement across organizations. As such, partners were involved in nearly all aspects of the activities such as selection of pilot priorities, participating in publications [20–23], grants, and presentation, and conducting research pilots. Additionally, within the Implementation Laboratory, BRIDGE-C2 has built capacity for Implementation Science in four main ways: building surveillance systems, improving recruitment and retention, clarifying data definitions, and increasing knowledge. The purpose of Laboratory surveillance activities is to collect monthly, real-time summaries of cancer screening and prevention rates (colorectal cancer screening, cervical cancer screening, HPV vaccination, and tobacco use and cessation), clinical characteristics, and HIT-driven interventions across the entire Laboratory in order to identify temporal associations between clinic performance on cancer preventive care delivery and specific HIT interventions. This information can be utilized to generate hypotheses for research studies, targeted sampling selection, and to suggest possible areas for exploration and intervention. The practice surveillance, developed with BRIDGE-C2 infrastructure funds, provides a ready sample of primary care clinics that can be stratified based on clinic characteristics as well as performance on cancer screening metrics [24].

## Domain 2: Clinical

The ISC3 initiative aimed to improve uptake of EBIs across the cancer continuum. All pilots conducted by BRIDGE-C2 target health equity and identify strategies to improve the uptake of EBIs. Our work had impact across all clinical subdomains (see Table 2). For example, several pilots have focused on addressing guideline changes and improving guideline adoption. Other pilots use technology and workflow redesign to implement EBIs and improve equity.

### Subdomain 2a: Procedures/guidelines

BRIDGE-C2 conducted a pilot evaluating the adoption and effectiveness of a cervical cancer screening clinical decision support (CDS) tool in the OCHIN Epic EHR. This pilot was intentionally designed to help our partners address guidelines changes around cervical cancer screening and follow up of abnormal pathology. This mixed methods pilot was conducted in partnership with OCHIN’s Clinical Operations and Improvement Teams to evaluate current usage of the tool while informing changes to optimize its functionality, improve adoption for patient care, and increase adherence to the new care guidelines. The pre-guideline adaptation tool was used in 41% of clinics [22]. Interviews with clinic staff indicated that low adoption was associated with lack of awareness of and training on the tool, poor data integration with relevant health maintenance (HM) topics, and complexity of the fillable form. These findings informed the creation of new training documents and user workflows inclusive of all relevant care-team members, a new HM topic allowing for easier abnormal result tracking, and a redesign of the EHR interface to better display the link to the CDS tool. We expect these changes to improve tool adoption in OCHIN health systems, especially follow-up of abnormal results, and thus improve patient outcomes.

### Subdomain 2b: Tools and products

BRIDGE-C2 conducted several pilots with impact on this subdomain. For example, the BRIDGE-C2 team alongside primary care clinicians in the OHSU Health network developed and implemented a multistrategy pilot targeting improvement in melanoma detection. This pilot developed workflows related to using technology to enhance skin cancer early detection and treatment in primary care. Two primary care clinicians identified a number of important barriers to skin cancer screening, including lack of knowledge, time, and clinical workflows to support routine screening and appropriate triage, and a priority need to implement strategies to improve melanoma detection. The pilot tests the feasibility of a multicomponent education intervention (group and online training, provision of smartphone dermatoscope device and EHR tools) for improving identification and triaging of skin cancer in primary care. The intervention was implemented in one FQHC and one rural health center in the BRIDGE-C2 Implementation Laboratory. Early findings suggest that the training improved primary care clinicians’ knowledge about skin cancer detection and increased the use of e-consults, but also that training for EHR tools was needed to support skin cancer detection, informing future adaptations of this intervention.

## Domain 3: Community

BRIDGE-C2, with its focus on partnership with community organizations, has had impacts across all subdomains (Table 3). Nearly all BRIDGE-C2 pilots had impacts on improved markers of health and/or reduced inequities, leading from the implementation of EBIs.

### Subdomain 3a: Health care delivery, health activities, and products

We developed and disseminated a pragmatic toolkit that focuses on implementation of a community-based vaccine site in a health center/clinic building equipped to provide clinical care [23]. This guide was rapidly developed to assist COVID-19 vaccine campaigns. This guide was developed in response to a need for creating a vaccine clinic in a primary care rural health center (OHSU Family Health Center in Scappoose, Oregon). The guide includes information for clinician regarding details on the specific vaccines and information they can provide their patients. The guide also provided extensive details on resources (staff, storage), workflow, space utilization, vaccination procedures (from order, storage, to documentation) to create a vaccine clinic. Although this toolkit is not specific to cancer, it is an emergency response to a crisis that impacted health equity and primary care.

### Subdomain 3b: Health care characteristics

Several pilots have conducted preliminary work that will lead to the rapid identification of the implementation strategies that are most effective at increasing the uptake of the EBI and at reducing inequities in accessing preventive cancer care. For example, in the Social Risk and Cancer Prevention pilot, we estimated the rates of breast, cervical, and colorectal cancer screenings among patient with social risk and found that patients with housing, food, and transportation insecurity had lower rates of screening. Using a survey, we also found that clinicians consider patients’ social circumstances when developing care plans. The clinicians reported interest in EHR tools that would help them tailor plans for these patients. This pilot showed the importance of collecting social risk data but also the need for tools that can assist clinician tailor their care plan based on the social need.

### Subdomain 3c: Health promotion

A potential impact of our work is increased uptake of preventive services and subsequently improved health and reduce inequities. As seen in Table 3, several pilots targeted improvement in cancer prevention EBIs. Two of these pilots targeted equity among gender minorities. One examined cervical cancer screening among transgender and gender diverse (TGD) individuals with a cervix who used testosterone therapy. Previous studies found that suppressed estrogen (a side effect of testosterone therapy) caused atrophic changes of the cervix leading to unsatisfactory cervical cytology results in this population. Our pilot assessed the frequency of inadequate and/or atrophic cervical cytology specimens among TGD patients undergoing testosterone therapy versus those not using testosterone. We conducted medical chart reviews of 213 patients identified as transgender patients with a cervix between 2012 and 2019. We found a relationship between testosterone usage and specimen inadequacy. We also found higher rates of missing transformation zone (transformation zone is the site of most intraepithelial neoplasia) in this population regardless of testosterone use. This finding warrants confirmation in other settings as it could impact guidelines for cervical cancer screening and HPV testing for this specific population.

## Conclusion

The modified TSBM was a helpful framework to categorize and describe the impacts of the BRIDGE-C2 Center. The team benefited from mapping four years of activities across a large center to the model, as it helped summarize progress and highlight and describe actual benefits. We also identified potential benefits that the team is now tracking for future projects. This retrospective evaluation showed that BRIDGE-C2 activities mainly centered on a few indicators, which aligned with the mission. Future initiatives may wish to use the TSBM from the beginning of the project as a planning and tracking tool to target all or specific indicators. BRIDGE-C2 provides a helpful case study to demonstrate empirical and real-world examples of how the TSBM can be applied in implementation science to show the impacts of research, community engagement, and capacity-building activities.

## Supporting information

Huguet et al. supplementary material Huguet et al. supplementary material

## Supplementary material

The supplementary material for this article can be found at https://doi.org/10.1017/cts.2024.682.
