# Supplementary material for: Impacts of pragmatic implementation science in a primary care laboratory
Source: J Clin Transl Sci. 2024 Dec 18;9(1):e20. doi: 10.1017/cts.2024.682 (PMC11795856; doi:10.1017/cts.2024.682)
Supplement: Huguet et al. supplementary material [file S2059866124006824sup001.docx]

| **Supplemental Table 1: Impact Indicators Added to the TSBM for Domain: Implementation Science** |
| --- |
| Subdomain: Implementation Science Methods and Measures |
| *Measures Development* |
| - Developed new measures of implementation determinants, processes, or outcomes |
| *Methods Development* |
| - Developed new methods for implementation strategy selection and optimization, or for identifying and prioritizing implementation determinants |
| - Developed new methods and toolkits for identifying and applying TMFs focused on health equity |
| - Conducted comprehensive summaries of existing methods and make recommendations for improvements |
| *-* Developed new engagement or co-creation strategies |
| - Developed pragmatic costing tools to inform decision makers and IS researchers |
| - Developed methods for assessing implementation and setting context |
| - Identified gaps in the literature |
| *Use of Rapid Cycle/Data Collection Strategies* |
| - Rapid needs assessment |
| - Used rapid cycle testing designs |
| *Adaptation* |
| *-* Developed pragmatic, low burden approaches to measuring adaptation, fidelity, and implementation cost |
| - Technological tools for tracking adaptations in clinical and community settings |
| - Function (vs. form) focused fidelity scales that are easily to administer in clinical and community settings |
| - Developed or adapted an implementation process or strategy with an explicit focus on health equity |
| *Developed methods for examining clinical/ community partner data in new ways/formats that supports their work* |
|  |
| ***Subdomain: Capacity-Building*** |
| *Building partner/practitioner research capacity* |
| - Partner led or participated on grants, publications, presentations |
| - Developed of partner’s own research infrastructure (e.g., pre- and post-award management, F&A, DUNS, biosketches) |
| - Developed partner skills in implementation processes |
| - Developed practitioner toolkits for integrating equity and/or costing into implementation science |
| - Developed tools to encourage the iterative use of IS frameworks to plan for, make midcourse adaptations during, and sustain EBPs in practice |
| - Developed tools to encourage the iterative use of IS frameworks by partners |
| *Engagement* |
| - Developed strategy for return of results to research partners and beyond; strategy is preferred by partner, relevant and actionable |
| - Partner included in selection of pilot grants |
| - Increased diversity of engaged partners |
| *Build IS research capacity* |
| - Included early investigators, trainees |
| - Increased diversity of investigator teams |
| - Increased skills of mentors |
| - Increased skills of early investigators and trainees at all levels |
| *-* Developed investigator toolkits for integrating equity into implementation science |
| - Extended IS efforts in the context of the partnership to new content areas in cancer control (e.g. climate change) |
| - Developed/Refined tools to aid in the planning of IS projects, selection, combination, adaptation, use and assessment of IS Theories, Models and Frameworks |
